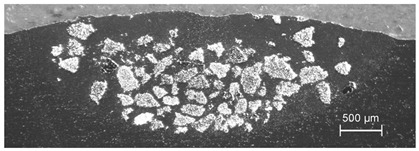# Wear Resistance Enhancement of Al6061 Alloy Surface Layer by Laser Dispersed Carbide Powders

**DOI:** 10.3390/ma13173683

**Published:** 2020-08-20

**Authors:** Rafał Jendrzejewski, Jacek Łubiński, Gerard Śliwiński

**Affiliations:** 1 Photophysics Department, Institute of Fluid Flow Machinery, Polish Academy of Sciences, Fiszera 14, 80-231 Gdańsk, Poland; gerards@imp.gda.pl; 2 Faculty of Mechanical Engineering, Gdańsk University of Technology, Narutowicza 11/12, 80-233 Gdańsk, Poland; jacek.lubinski@pg.edu.pl

**Keywords:** surface modification, laser dispersing, metal matrix composite, wear resistance

## Abstract

In this paper, results of the experimental study on improving wear resistance in sliding friction of Al-based alloy are presented. The technique used involves the formation of a metal matrix composite (MMC) in the alloy surface layer by laser dispersion of carbide powders such as WC, TiC and SiC. For WC and TiC MMC surface coatings fabricated under conditions typical for most of the technologically relevant solid-state lasers (wavelength range of 0.8–1.1 μm), the nearly inversely proportional dependence of the required laser energy density on the powder mass density is observed. Highly homogenous distribution of powder particle content (up to 40%) in the MMC surface coatings of a thickness between 0.8 and 1.6 mm obtained by multiple scanning is observed in the cross-section of specimens processed within a rather narrow parameter window. Tribological tests and comparison to untreated material reveal wear resistance increases by five- and ten-fold, observed in samples with laser-dispersed TiC and WC powders, respectively. Results indicate that substantial modification and reinforcement of the surface layer can be achieved in Al alloy in a one-step process without substrate preheating.

## 1. Introduction

Advantages of light metal alloys in various areas of engineering and construction are obvious; the low density combined with acceptable strength makes production of lightweight structural components possible, while maintaining the capacity to carry the load at a desired level. Such combination fulfils the critical requirements in most designs, often making light metal alloys a competitive choice against steel, even at greater cost. A downside of most aluminum, magnesium and titanium alloys is their inferior resistance to wear in sliding friction compared to steel, which hampers their potential use in many applications. Overcoming that downside of the light metal alloy component by a widespread use of reinforcing treatment of the surface and sub surface zone could be the important breakthrough in broadened application of the entire group of materials. A promising way of achieving such a goal is the use of high intensity heat sources to selectively melt the near-surface layer, inflicting a change in the structure and/or composition of the layer.

Among methods of the surface structure modification and wear resistance enhancement of light metal alloys, interesting results have been obtained with use of the intense pulse electron beam as reported by Ivanov et al. for the case of silumin (Al–Si alloy) [1]. In the case of electron beam treatment (EBT), melting of the silumin surface layer results in dissolution of Si, inclusions and intermetallic compounds, and leads to the formation of a structure characterized by cellular crystallization with reprecipitated submicrometer- and nano-sized particles.

A novel, recently discussed perspective in this field is associated with a broader use and processing potential of high power laser tools. Extremely high energy density at the surface of the processed object offered by lasers allows for the treatment of any material, even ceramics. Innovative surface treatments such as laser texturing can be applied to ceramic coatings (Al_2_O_3_–13%TiO_2_) [2] on steel substrate, and also Ti6Al4V alloy [3,4]. Results indicate markedly improved tribological performance characterized by changes in the roughness and structure. In addition, improved lubricant retention and surface wettability were observed. It is worth mentioning that laser technology can also be used in additive manufacturing (LB-PBF—laser beam powder bed fusion or SLM—selective laser melting), yielding tribological properties superior to the bulk material and beneficial for the performance of the machine components produced [5]. Furthermore, a successful production of light metal and ceramic powder-based Al+SiC_p_ composites has been reported, manufactured by laser metal deposition (LMD)—an additive manufacturing process which allows for the obtainment of metal—ceramic composite specimens exhibiting high resistance to wear [6].

The versatility of laser treatment can be illustrated by improvement of the tribological characteristics in a controlled manner reported by Dilawary et al. [7], who applied laser treatment to the plasma hard coating (ceramics) and observed a modified and refined crystalline structure of the composite surface layer. For a similar processing of the reduction in pores, cracks and a lamellar structure contributing to improved homogeneity of the coating were reported [8]. The observed enhancement of wear resistance was assigned to the change of the wear mechanism from spalling to micro-cracking.

Formation of new phases capable of transforming into tribo-films in sliding friction was reported for laser surface remelting applied to metallic materials (e.g., Inconel 600, equivalent to NiCr15Fe/2.4816) to locally alloy the substrate (B and CaF_2_) [9]. Such treatment shows an anti-friction and anti-wear potential resulting from the presence of a solid lubrication film formed within the frictional contact zone. Another surface treatment of texturing by laser was applied to industrial grade TiN and WC/C coatings allowed for wear resistance improvement in sliding (oil lubricated) thanks to micro-pocket formation and lubricant retention in the friction zone [10]. In their extensive work on rail tribology, Roy et al. [11] developed a surface repair treatment for high contact stress-loaded components, such as steel rails, by laser cladding 410L (X2CrNi12/1.4003), SS420 (X20Cr13/1.4021) or Stellite 6 coatings. Such repair alloys of high tensile strength represent a promising technology in railway maintenance and repair, anti-fatigue and wear treatment on new rails [12] and simulations were also performed of contact overheating of rail wheels on a model tribological contact by means of pulsed laser heating in surface quenching treatment [13]. Among ongoing studies on control of the tribological characteristics of sliding or rolling contacts by means of laser technology, these regarding light alloys are relatively scarce—except for the field of laser dispersing into aluminum- and titanium-based materials.

**Figure 1:**
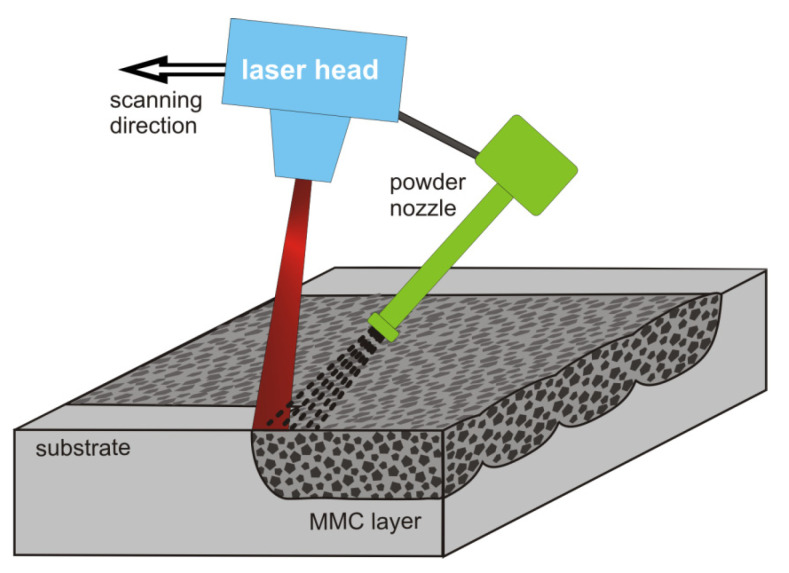
A schematic drawing of the principle of formation of the MMC layer by the laser dispersing process.

Among laser-based surface treatment techniques, which can be applied to light alloys, the laser dispersing (also called laser embedding or laser melt injection) offers a promising perspective of high wear resistance. The process is a combination of local melting of the metal by a laser beam, while simultaneously blasting the melted spot with a high velocity stream of micro grains (powder) of hard compound. Due to high kinetic energy, the powder grains can penetrate the melted pool of substrate to form a metal matrix composite (MMC) layer through to the depth of the actual melt (Figure 1).

The technique is a form of laser alloying. The sub-surface layer of multi-phase material exhibits significantly increased strength to density ratio, compared to the untreated volume. Parameters of laser injection of hard particles must be optimized for a successful treatment. It is critically important to achieve the desired depth of melt while avoiding excessive melting of the powder particles as they transit the laser beam. One of the great advantages of laser dispersing is the exact control over the location of the treated area. The MMC layer can be applied precisely at the required location. The technique also offers the added capacity to facilitate functionally graded materials (FGM), in which the properties change with depth into the treated surface [14,15]. Comparatively, laser hardening and cladding prove to be inferior. However, for the effect observed in the case of the worn surface of laser-cladded MMC coatings consisting of TiC powder grains dispersed in Al-Si alloy (12 wt% Si) substrate, a material removal mechanism was postulated involving crack propagation leading eventually to debonding of the tribo-layer fragments [16].

For laser dispersing of SiC powder in Al alloys, some results indicate consistently the narrow window of allowable processing parameters due to oxide skin formation on the Al surface [14,17,18,19] and for TiC powder dispersing into AlSi7 (AC–AlSi7Mg) alloy; also, the influence of the laser intensity distribution was observed [20]. Recently, for the Al6061 substrate, the combined wire and powder deposition by laser [21] and also selective laser melting of pure Al and Al–Cu–Fe–Cr powder grains for production of reinforced MMC structures with post-aging treatment [22] were reported. These works, including also our previous reports on the dispersing of SiC powder grains into Al6061 (AW–AlMg1SiCu) alloy [23,24] and SiC [25] and WC or TiC [26] powders (mainly in Ti6Al4V alloy), indicate the growing interest and confirm the research efforts in laser dispersing. However, plenty of the studies were performed for different substrates, various dispersed powder materials, and with laser sources of various characteristics—see Table 1 where data for the case of Al-based alloys are collected showing the novelty of this work too.

Notably, the reported laser parameters differ regarding the operating wavelength and the beam intensity distribution which affect substantially the processing through the wavelength dependent material absorption and heat distribution homogeneity due to laser irradiation, respectively. As the use of near-infrared lasers is preferred, due to acceptable absorptivity of light metal alloys in the wavelength range of 800–1100 nm, further systematic studies with these laser tools are advisable with regard to focused research advancement and application potential.

In this paper, the wear resistance enhancement resulting from structural surface modification due to laser-dispersed carbide powders in the aluminum alloy Al6061 is reported. Al6061 was selected as substrate because it represents a useful platform for the systematic study of dispersing effects as concluded from previous reports. Compared to a substantial part of the latter, no preheating of the substrate prior laser dispersing was assumed in the present work in order to investigate conditions for the cost and time-saving one-step MMC fabrication. Moreover, using the pulsed, ytterbium-doped yttrium aluminium garnet (Yb:YAG) laser ensured irradiation characteristics typical for a broader group of technologically matured slab, disc and fiber solid state lasers operating in the near-infrared wavelength range around one micrometer, as can be concluded from the data in Table 1. The process parameters were studied for three different carbide powders SiC, TiC and WC and the substantially improved tribological characteristics are discussed based on microscope inspection, chemical analysis and wear testing.

## 2. Materials and Methods

### 2.1. Laser Stand

The experimental stand used in this work was equipped with an 8 kW Yb:YAG disk laser TruDisk 8002 (Trumpf, Ditzingen, Germany) operating at a wavelength of 1030 nm. The focal length of the beam forming optical lens was equal to 223 mm and the laser beam intensity profile was close to “top-hat”. The movement of the beam while processing the specimens was facilitated by a multi-axial TruLaser Robot 5020 (Trumpf, Ditzingen, Germany) carrying the working head of the optical system. The head was tilted off perpendicularly to the specimen surface by a 15° angle to avoid back propagation of radiation into the optical fiber and laser resonator. Details of the experimental set-up presented schematically in Figure 1 have been described in previous works [23,24,25,26].

### 2.2. Substrate Material and Reinforcing Powders

The alloy Al6061 (T651 temper, solutionized, stress-relieved stretched and artificially aged) used in this study was obtained from Aircraft Materials (Stokenchurch, UK), and the powders SiC, TiC and WC from Stanchem (Lublin, Poland), Changsha Langfeng Metallic Material (Changsha, China) and Technology Management Consultants (Włosań, Poland), respectively. Basic physicochemical characteristics of the materials are given in Table 2 (substrate) and Table 3 (powders). The selection of the carbide powder materials was substantiated by their high melting temperature *T_m_* as compared to light metal alloys, which prevent the particles from melting while transiting the laser beam. However, absorption of these powder materials is higher than that of the applied substrate at the applied laser wavelength, i.e., carbides can absorb up to about 90% of the irradiation energy [14,17]. Moreover, poor wetting of carbides by melted aluminum alloy affects negatively a proper injection. These factors, fundamentally important to the injection process, require joint tuning of the following parameters: (i) time of exposure of the substrate surface and powder particles to laser radiation, (ii) solidification time of the melted substrate after particle injection, (iii) velocities of scanning and of the particle stream (to achieve the desired content of powder in the matrix), and (iv) location of the powder injector nozzle relative to both the laser beam and the melted spot.

### 2.3. Powder Feeding

A lateral powder nozzle was applied to reduce the exposure of powder particles to laser radiation. It prevented activation of particles for diffusive and chemical interaction with the substrate material and formation of inter-material phases due to laser heating. The powder feeder and nozzle, using argon gas as propelling medium, were attached to the laser head. The same gas was supplied as a shielding agent expelling air from the melt zone. By controlling the gas flow rate through both powder and shielding gas nozzles, powder feed rate and particles’ velocity were adjusted. The nozzle was inclined by 45° against the substrate top plane (at 30° in relation to the laser beam axis). The position of the powder jet impact spot on the specimen relative to the laser irradiated one was adjustable. It was assumed that the most homogeneous distribution of powder grains would be achieved with the injection area trailing behind the irradiated spot, compared to overlapping of irradiated and impact spots. This no-overlap configuration ensures no radiation heating (and melting) of powder at the cost of elongation of the melted pool, which required more laser power applied.

**Figure 2:**
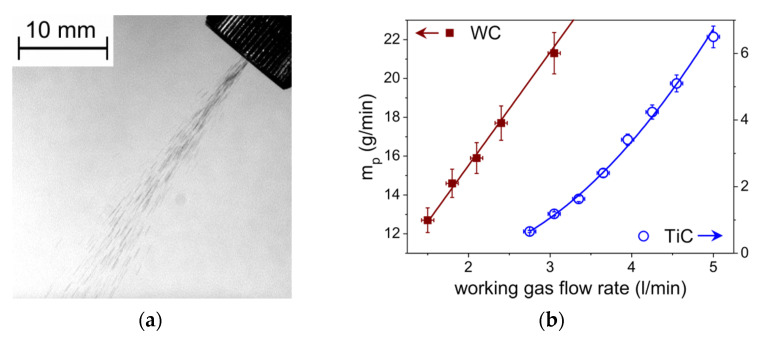
(**a**) Photograph of the WC powder stream (exposure time 1 ms); (**b**) Dependencies of powder feed-rate vs. working gas flow rate for TiC and WC powders.

As the quality of the process was found to be strongly dependent on the particle distribution in the powder stream, the initial tests were performed with a photographic recording of the powder jet to visualize its structure on the nozzle exit. From the observation of the flow rate effect of carrier gas on the powder feed rate and velocity of particles, the powder feed-rate was found to be proportional to the gas flow (Figure 2). Optimal distance between the substrate surface and nozzle, ensuring saturation of the entire melted zone with powder particles, was obtained from visual inspection of the particle impact area. It was observed in agreement with the literature that both the volume of the melted pool and particle embedding depth correlate with the particle velocity, which decreases with distance from nozzle exit and, less observably, with scanning speed [14,17,27].

### 2.4. Selection of Process Parameters

The results of laser dispersing can only be observed in an ex post examination of a cross-section of the produced trace(s). Therefore, despite the fact that the aim of this work was to obtain the surface layers consisting of several tracks, first an extensive set of single trace tests was necessary to find the range of the available process parameters—see Table 4 where the laser energy density is defined as *E*_0_ = 4*P*/(π*d_l_v_sc_*). The preparation phase was based on similar research experience of our own [23,24,26] and external [14,17,18,19] origin, which helped estimate the general envelope of operation. However, the final parameters’ selection had to be individualized by tests performed in the actual laser system used in this research. Presumptions were made of no preheating of the substrate and the desired effect being a modified layer of at least 0.5 mm in thickness, homogenously saturated with powder particles.

The useful range of parameters identified in single trace tests was applied for the selection of fabrication parameters of MMC specimens in the scanning/multi-trace mode to obtain composite coatings comprising several parallel, overlapping tracks reinforcing the base material. The viable range of process parameters, as evaluated in single track tests, was fully covered (scanning speeds, powder feed rates, laser beam intensities and different geometries of the experimental set-up) and the final examination of results allowed us to select the most effective combinations of process parameters in multi-trace mode. Depending on the laser spot on the sample surface *d*, the number of traces *n* (11–18) and the distance between adjacent paths Δ*x* (1.8–3 mm) were selected in order to cover a square shape area of approximately 35 mm × 35 mm. Due to the lateral powder nozzle used, the forward direction of laser head movement was active with return stroke idle (no lasing) while scanning. After each return movement, the head was shifted sideways by the same increment of the last track covered. The reposition time of the laser head was sufficient to decrease the temperature gradients and to prevent crack formation in the specimen before the forming of the next track was commenced.

## 3. Results and Discussion

For single and multi-track MMC samples inspected visually and exhibiting the powder grains firmly embedded in the processed surface, metallographic specimens were prepared and studied by means of the optical and scanning electron microscopy (SEM, Zeiss EVO 40, Oberkochen, Germany). The SEM inspection was accompanied by measurement of the energy-dispersive spectra (EDS) using the Quantax 400 spectrometer with SDD X-flash 5010 detector (Bruker, Berlin, Germany) to determine the elemental composition in the sample cross-sections.

### 3.1. Single Track MMC Layers

For the dispersing experiment, first the substrate melting by laser beam was investigated. It was observed that for the alloy Al6061, the most efficient surface melting could be obtained in a relatively narrow range of processing parameters, i.e., *v_sc_* = 5–15 mm/s, and *I* = 260–530 W/mm^2^ defined for two laser spot size values of 2.5 and 3.1 mm. Under such conditions, the depth and width of melted zones were within the range between 0.8 and 1.6 mm, and 3.7 and 5.1 mm, respectively. Such dimensions ensured that the MMC surface layer had enough excess material to grind flat the specimens for further investigation.

**Figure 3:**
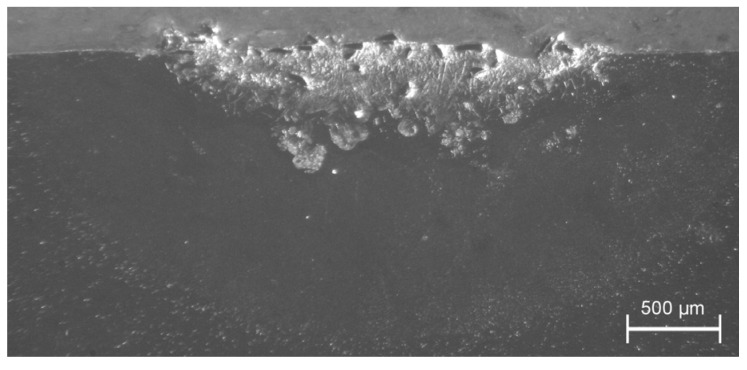
Laser dispersed SiC powder into Al6061 alloy: partially melted powder particles are accumulated in the top layer of the melted zone; processing parameters: *E*_0_ = 217 J/mm^2^, *v_sc_* = 7 mm/s, *m_p_* = 1.3 g/min.

In the case of composite traces with SiC powder, the depth ratio of the grain penetration to that of the melting zone was unacceptably small and also melted SiC particles were observed—see Figure 3. This is in accordance with our own experience and literature data, indicating that the fabrication of a composite layer of the Al-based alloy and SiC materials pair requires the substrate preheating to approximately 300 °C [14,17,23,24] or enhancement of the laser processing by the tungsten inert gas (TIG) arc method—a technique originally invented for the WC powder [28].

The process parameters for TiC powder and corresponding cross-section photographs are summarized in Table 5. The cross-section of sample 1 reveals a small number of injected TiC grains, located mainly in the bottom layer of the molten substrate, an effect reported mainly for dispersing of the heavier WC particles [29]. The increase in laser beam intensity by decreasing the laser beam spot resulted in a higher number of injected particles, though localized in the same way (sample 2). Using the parameter set as for sample 1 and corrected positioning of the powder nozzle resulted only in an insignificant improvement of the particle distribution (sample 3). Increased number of trapped grains in the lower half of the melted zone was observed for reduced *v_sc_* (sample 4). At this *v_sc_* value, the best dispersing results were obtained for increased laser beam power, corresponding to an energy density of 329 J/mm^2^ (sample 5) and the distribution of the particles in the whole melted zone was close to homogenous, despite the irregular shape of the TiC powder grains. The TiC-enriched part of the track covered approximately 85% of the melted zone width and the particles were injected to an acceptable depth of about 1 mm. The TiC volume fraction in the melted substrate area, here and hereafter estimated by means of the ImageJ software, was equal to about 35–40%. The observed result obtained at low scanning velocity corresponds with results of laser dispersing of light carbide powders (TiC, SiC) into aluminum alloys reported in the literature [14,17,20,23,24]. For sample 6—fabricated at increased scanning speed and powder feeding, but reduced laser power—the TiC volume fraction in the melted zone exceeded 40% after mechanical removal of about 500 μm of the substrate top layer scarcely saturated with injected particles.

**Figure 4:**
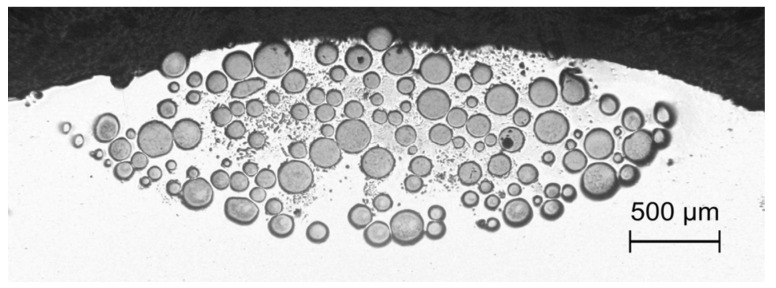
Cross-section of composite trace in Al6061alloy with WC powder particles; experimental conditions: *E*_0_ = 82 J/mm^2^, *v_sc_* = 10 mm/s, *m_p_* = 30 g/min.

In the case of WC powder dispersing, the MMC traces were produced at powder feed rates in the range of 15.9–30 g/min and results obtained for the pair of materials Al6061 and WC are discussed in more detail elsewhere [26]. Those results indicated that, in comparison to dispersing of TiC powder, an increase in the scanning speed up to 15 mm/s was possible at laser energy density reduced to the range of 55–82 J/mm^2^. This was due to the much higher mass density of this powder material (see Table 3) resulting in a correspondingly higher kinetic energy of the WC powder grains which allowed for an easier overcoming of the oxide skin barrier and depth penetration of the melted substrate. In the WC enriched tracks (see Figure 4), the reinforcing material was homogeneously distributed in the melted area, the trace width with embedded WC grains reached 80–85% of the melted path width and the filling factor in the melting zone was between 30 and 40%. Notably, the final effect of surface reinforcement in specimens with higher concentration of the injected powder in the bottom layer of the melted zone can be improved by grinding off the top layer containing only a scarce number of hard particles.

**Figure 5:**
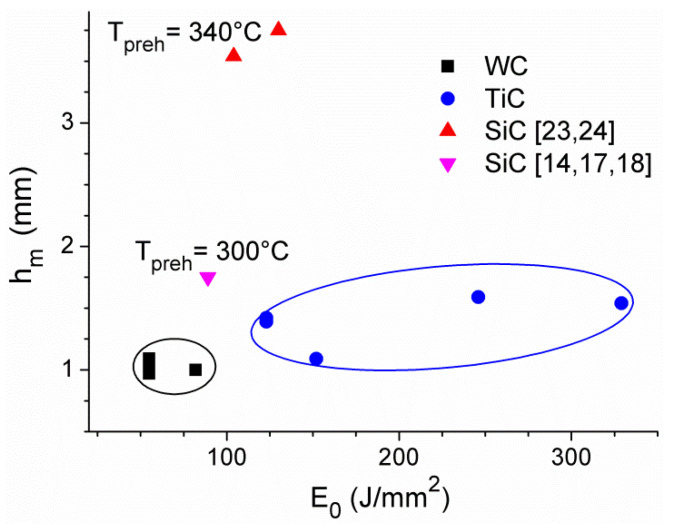
Parameters of the laser dispersing of carbide powders in Al-based substrates, results of this work and literature data shown as dependence of the melting depth *h_m_* vs. laser energy density *E*_0_, with assumed homogenous grain distribution; the MMC single trace width is at least 85% that of the laser spot in all cases except that of SiC powder dispersed in Al preheated to 300 °C (pink down-pointing triangle).

The parameters obtained here for dispersing WC and TiC powders in Al6061 alloy by means of Yb:YAG laser together with these for SiC in Al-based substrates obtained by means of Nd:YAG [14,17,18] and high power diode lasers [23,24], operating in the wavelength range of 800–1100 nm and providing similar processing conditions, are summarized in Figure 5. It should be noted that results for SiC dispersing were obtained with substrates preheated to 300 °C (Nd:YAG laser case) and 340 °C (diode laser case), and that in the first case results for the single track of the width not exceeding 40% of the laser spot size were reported only, while for other results in Figure 5 this value was not less than 85%. The observed distribution of results is in part due to differences in experimental conditions, including the possible effect of the spatial intensity profile of the laser beam. The collected data indicate that in dispersing heavier powders (WC), acceptable technological results characterized by homogenous grain distribution in the melted volume of single traces wider than 85% of the laser spot size and of maximum depth *h_m_* in the range of 0.9–1.1 mm can be obtained for a relatively narrow optimal range of the laser energy density (55–82 J/mm^2^), see Figure 5. The respective selection of parameters coupled in *E*_0_ (scanning speed and laser beam power/intensity) is limited by laser capabilities and the characteristics of the powder nozzle. Interestingly, for the relatively light TiC powder comparable technological results require values of *E*_0_ almost two times higher than observed for WC dispersing with use of the same laser tool. This means, however, the necessity of a suitable increase in the laser beam intensity and/or reduction in the scanning speed, thus resulting in less efficient production, and increased range of *h_m_* (1.1–1.6 mm). Moreover, despite the known negative oxidation effect in Al-based alloys, for dispersing of the relatively lighter SiC grains the one-step process (without preheating) at higher *E*_0_ values, i.e., higher laser beam power and/or lower scanning speed, could also be considered.

### 3.2. Multi-Track Reinforced MMC Coatings

**Figure 6:**
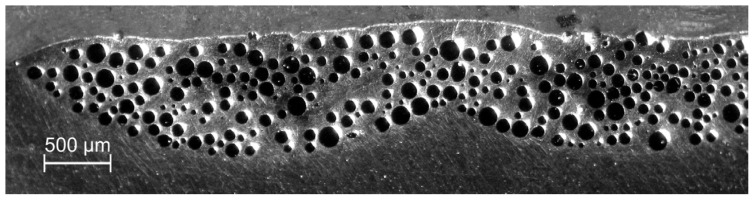
Cross-section of the multi-trace MMC Al6061 + WC_p_ surface layer; experimental conditions: *I* = 260 W/mm^2^, *v_sc_* = 10 mm/s, *m_p_* = 30 g/min.

MMC surface coatings containing WC particles dispersed in several adjacent traces and exhibiting the most homogeneous particle distribution in the visual inspection were produced at *v_sc_* = 10 mm/s, *I* = 260 W/mm^2^, *d* = 3.1 mm, Δ*x* = 3 mm and *m_p_* = 30 g/min. The cross-section shown in Figure 6 reveals a uniform distribution of WC grains throughout the surface layer, with the melting/dispersing depths reaching the values from 0.6 mm in the overlapping regions of two adjacent traces, to 0.9 mm along the track centerlines. This narrowing of the processing depth is ascribed to the laser intensity distribution in the spot and heat transfer in the material induced by the treatment. It is worth mentioning that for samples revealing a small amount of grains in the upper part of the melted volume, the mechanical partial removal of this part (0.5–0.7 mm in thickness) has uncovered the deeper zones with sufficiently high concentration of embedded powder, advantageous from the application point of view.

**Figure 7:**
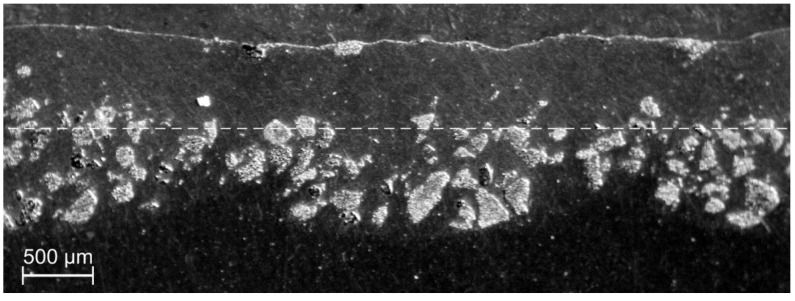
Cross-section through a composite layer based on Al6061 alloy with TiC powder; experimental conditions: *I* = 400 W/mm^2^, *v_sc_* = 10 mm/s, *m_p_* = 6 g/min.

In the TiC dispersing, the qualitatively best results were obtained at *v_sc_* in the range of 6–10 mm/s for *m_p_* = 6 g/min and *I* = 400 W/mm^2^, while the laser spot was 3.1 mm, and the shift between consecutive traces was equal to 2.8 mm. As observed in Figure 7, the injected material is distributed evenly at the bottom of the melted area characterized by the melting depth varying from 0.9 mm at joints of the traces to 1.4 mm along the centerlines. The post-treatment grinding (approximately 0.6 mm deep, see dashed line in Figure 7) could result in the remaining composite layer of thickness between 0.3 and 0.8 mm and the estimated filling factor of the TiC powder of 35%. An even more uniform filling of the melted volume can be expected for higher *m_p_* value and lower scanning speed.

### 3.3. MMC Morfology and Chemical Composition

**Figure 8:**
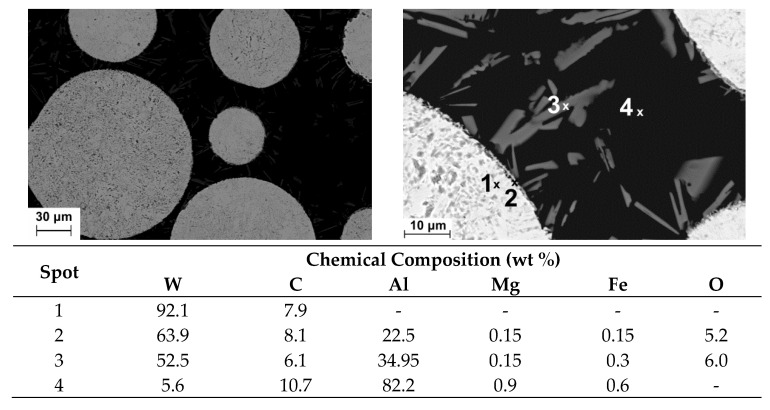
SEM photographs of the Al6061 + WC MMC layer fabricated under conditions the same as in Figure 4, and chemical composition obtained at selected spots.

The EDS data of the WC powder dispersion in Al 6061 were collected for the distinctly visible powder grains, Al matrix and needle-like precipitates observed in the grain interspaces at higher magnifications—see Figure 8. Similar precipitates were also evidenced in MMC layers produced with TiC powder, again mainly in the vicinity of the powder particles. Data collected in Figure 8 indicate the presence of carbon (spot 3), which can be associated with Al_4_C_3_ platelets in agreement with the literature [14,17,18,30]. Formation of these platelets is attributed to the dissolution of carbide powders by the laser beam and/or contact of the grains with the liquid aluminum matrix. It is also confirmed by EDS results obtained for the powder grains, where the thin layer of the particle (spot 2) reveals chemical composition different from the bulk of the grain (spot 1). The primary oxide skin on the WC grains (spot 2) and its dissolving during the laser processing are the most likely cause of the oxygen presence in the needle-like structures (spot 3), as the use of argon shielding should eliminate the atmospheric oxygen ingress. Chemical composition of the matrix (spot 4) is close to that of the original Al6061 substrate with a small amount of dissolved carbon and tungsten.

### 3.4. Wear Resistance

The wear resistance was measured for representative samples using the “ball-on-disc” method designed to measure wear at a constant load, with the flat surface of the MMC sample acting as a rotating disk in a sliding contact against fixed Al_2_O_3_ balls of 5 mm dia. Prior to measurements, samples were ground-down, polished, thoroughly washed, degreased and dried. The load of 26 N was applied to a non-rotating ball and sliding velocity was kept constant at 0.1 ± 0.02 m/s. Tests were performed without lubrication under ambient conditions, at a relative humidity below 40%. The test parameters used were initially estimated on the basis of earlier experience [24] and fine-tuned in preliminary testing. The use of a ceramic ball instead of 100Cr6/1.3505 steel was necessitated by extremely rapid wear of the steel ball observed in sliding contact with the modified layers (especially with WC). The load–velocity combination is a compromise between wear rate feasibility for the test program and acceptably low temperature of the specimen to avoid excessive oxidation on the surface of the aluminum alloy. The temperature increase in the bulk did not exceed 10 °C for the total sliding distance between 200 and 1000 m, depending on ball wear. Data for the wear quantification were collected at 100 m sliding distance intervals by a volume loss calculation based on the averaged multiple profilometer scans. For reference, data were collected both for samples of untreated alloy material and those treated by laser without powder injected under conditions similar to the dispersing process. Two wear tests were made for each sample and the measurement data were averaged.

**Figure 9:**
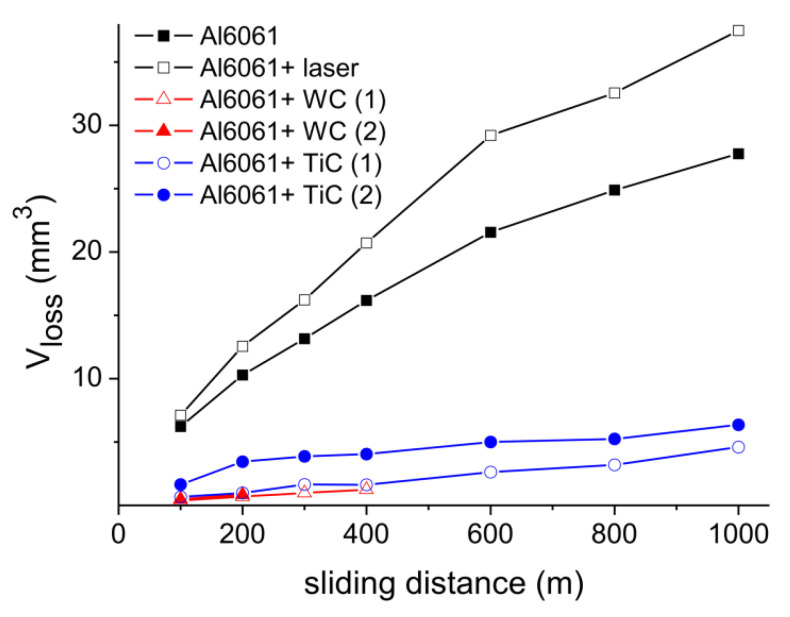
Volume losses calculated for investigated samples: reference Al6061, reference laser-treated alloy, two MMC layers with injected WC powder and two Al6061 + TiC samples made in different experimental conditions.

Results summarized in Figure 9 reveal a close to linear dependence of the volume loss in investigated samples on sliding distance. Such a result confirms a uniform distribution of the reinforcing material as a function of the MMC depth. While for TiC the long sliding distances were fairly reproduced in consecutive measurements, the relatively shorter sliding distances observed in the case of WC-enriched layers resulted from a rapid wear of the testing balls. Interestingly, the laser irradiated substrate exhibited volume loss larger than the non-irradiated one, which can be explained with a substantial amount of oxygen penetrating the remelted layer and the formation of Al oxides.

**Figure 10:**
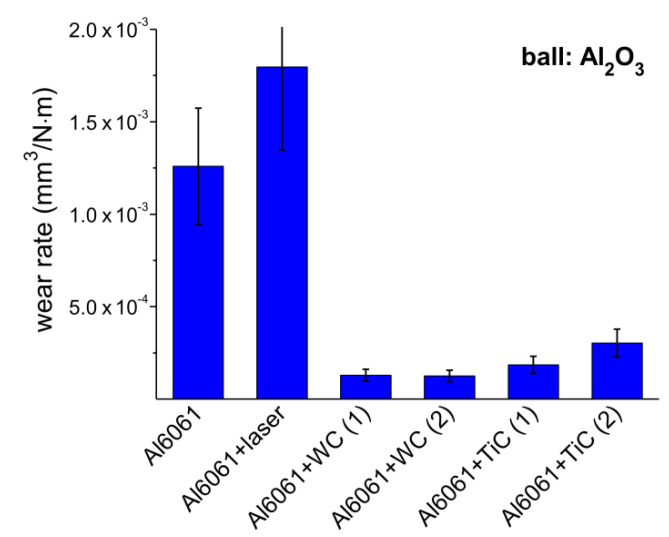
Wear rates calculated from the volume loss vs. sliding distance dependencies shown in Figure 9.

The averaged wear rate values, corresponding to volume loss divided by the sliding distance and the applied load, are presented in Figure 10. Slight differences observed between the results in consecutive measurements on the same material pairs are inevitable due to the experimental conditions, including also the wear testing in dry sliding friction. Especially in cases of surface treated materials with inherent non-homogenous, multiple-track structure with cross-track changes of the material concentration, the resulting measurement errors of up to 25% can be expected. Even with these factors taken into account, the data comparison indicates impressive differences between reference materials and reinforced samples. The latter exhibit, in the case of TiC and WC powder-reinforced alloy Al6061, the values of 2.5 × 10^−4^ and 1.3 × 10^−4^ mm^3^/(N·m), respectively. Wear rates observed for reference samples are greater than those of Al6061 + WC MMCs by nearly ten and fourteen-fold in the cases of the untreated and laser-treated Al substrate, respectively. Both the substantially improved wear rates resulting from the presence of hard phase grains strongly coupled to the matrix, and the homogeneous MMC microstructures obtained by the careful selection of the laser dispersing parameters confirm consistently the application capacity of the one-step process implemented. It is noteworthy that a comparable level of wear resistance improvement has been reported for SiC reinforced MMC layers on the substrates of Al6061 [23] and Al–8Si [19].

## 4. Conclusions

Parameters of the laser dispersing of SiC, TiC and WC powder particles into Al 6061 alloy, such as the laser beam intensity and interaction geometry, scanning speed, and powder feed rate were experimentally investigated and their influence on properties of MMC surface layers, including microstructure, chemical composition and wear resistance, was studied. For two of the three investigated powders, WC and TiC, dispersed with use of a 1030 nm Yb:YAG laser, optimal process parameters were found, and surface layers with homogeneously dispersed powder material were fabricated in a one-step process, without preheating of the Al substrate. Laser dispersing of heavier WC particles into Al6061 alloy was found to require lower laser beam intensities, compared to the case of the much lighter TiC, in which a reduction in scanning speed was also necessary. This indicates that the required laser energy density is nearly inversely proportional to the powder mass density. Most importantly, the wear tests showed significant improvement in abrasion resistance of obtained composite surfaces compared to bulk alloy material. Wear rates of surfaces with injected TiC and WC powder grains were 20% and 10%, respectively, of those obtained for reference samples.

Results from this study confirm that laser dispersing using carbide powders represents a promising method of producing wear-resistant composite materials based on light metal alloys, such as aluminum-based ones. It is expected, that the barrier in industrial application of this method, due to low productivity resulting from relatively low scanning speed, will soon be overcome with the high-power solid-state lasers coming into age. The present study indicates that in special applications requiring a locally reinforced sliding surface of a light alloy component, the laser-assisted hard powder dispersing is a feasible technology offering low invasiveness and competitively high gain in wear resistance.

## Figures and Tables

**Table 1:** Literature data on laser dispersing of carbide powders in Al-based alloys.

Powder	Alloy	Laser	Wavelength (nm)	Beam Profile	Substrate Preheating	Single/Multi-Tracks	References
SiC	Pure Al	Nd:YAG	1064	Gaussian	Yes	Single	[14,17,18]
SiC	Al–8Si	Nd:YAG	1064	Gaussian	Yes	Single	[19]
TiC	AlSi7	CO_2_	10,600	Multimode	No data	Single	[20]
SiC	Al6061	Diode	808 and 940	Top-hat	Yes	Multi	[23,24]
TiC, WC	Al6061	Yb:YAG	1030	Top-hat	No	Single	[26]
SiC, TiC, WC	Al6061	Yb:YAG	1030	Top-hat	No	Multi	This work

**Table 2:** Physicochemical properties of the alloy used in the experiments.

Alloy	Chemical Composition	*ρ* (kg/m^3^)	*T_m_* (°C)
Al6061	0.8–1.2% Mg, ≤0.7% Fe, 0.4–0.8% Si, 0.15–0.4% Cu, ≤0.25% Zn, ≤0.15% Mn, 0.04–0.35% Cr, ≤0.15% Ti, bal. Al	2700	585

**Table 3:** Main physical properties of powders used in the laser dispersing studies.

Powder	Grain Shape	Grain Size (µm)	*ρ* (kg/m^3^)	*T_m_* (°C)
SiC	Irregular	~80 (60–100)	3200	2730
TiC	Irregular	~95 (50–150)	4900	3160
WC	Spherical	~100 (45–150)	16,400	2870

**Table 4:** Process parameters of the single trace MMC fabrication.

	Scanning Velocity *v_sc_* (mm/s)	Powder Feed-Rate *m_p_* (g/min)			Laser Power *P* (W)	Laser Beam Spot Size *d_l_* (mm)	Laser Beam Intensity *I* (W/mm^2^)	Laser Energy Density *E*_0_ (J/mm^2^)
		TiC	WC	SiC				
Min	5	1.6	15.9	1.3	800	1.3	110	33
Max	30	6.5	30	4	5000	3.1	2810	329

**Table 5:** Process parameters and microstructures of single MMC traces of laser dispersed TiC particles in Al6061.

No.	*E*_0_ (J/mm^2^)	*v_sc_* (mm/s)	*m_p_* (g/min)	Sample Cross-Section
1	123	10	5.0	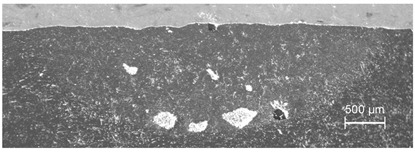
2	152	10	5.0	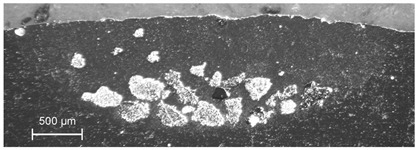
3	123	10	5.0	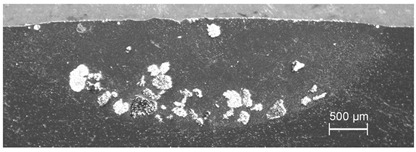
4	246	5	5.0	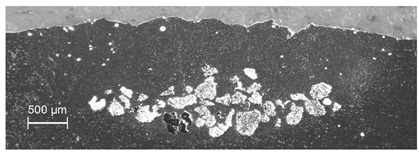
5	329	5	5.0	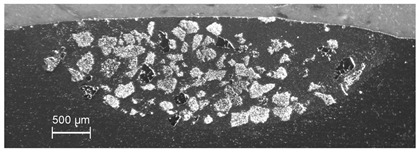
6	123	10	6.5